# Immunological Effects of Histotripsy for Cancer Therapy

**DOI:** 10.3389/fonc.2021.681629

**Published:** 2021-05-31

**Authors:** Alissa Hendricks-Wenger, Ruby Hutchison, Eli Vlaisavljevich, Irving Coy Allen

**Affiliations:** ^1^ Graduate Program in Translational Biology, Medicine and Health, Virginia Tech, Roanoke, VA, United States; ^2^ Department of Biomedical Sciences and Pathobiology, Virginia-Maryland College of Veterinary Medicine, Blacksburg, VA, United States; ^3^ Department of Biomedical Engineering and Mechanics, Virginia Tech, Blacksburg, VA, United States; ^4^ Institute for Critical Technology and Applied Sciences Center for Engineered Health, Virginia Tech, Blacksburg, VA, United States; ^5^ Department of Basic Science Education, Virginia Tech Carilion School of Medicine, Roanoke, VA, United States

**Keywords:** abscopal effect, histotripsy, focused ultrasound, immunomodulation, ablation

## Abstract

Cancer is the second leading cause of death worldwide despite major advancements in diagnosis and therapy over the past century. One of the most debilitating aspects of cancer is the burden brought on by metastatic disease. Therefore, an ideal treatment protocol would address not only debulking larger primary tumors but also circulating tumor cells and distant metastases. To address this need, the use of immune modulating therapies has become a pillar in the oncology armamentarium. A therapeutic option that has recently emerged is the use of focal ablation therapies that can destroy a tumor through various physical or mechanical mechanisms and release a cellular lysate with the potential to stimulate an immune response. Histotripsy is a non-invasive, non-ionizing, non-thermal, ultrasound guided ablation technology that has shown promise over the past decade as a debulking therapy. As histotripsy therapies have developed, the full picture of the accompanying immune response has revealed a wide range of immunogenic mechanisms that include DAMP and anti-tumor mediator release, changes in local cellular immune populations, development of a systemic immune response, and therapeutic synergism with the inclusion of checkpoint inhibitor therapies. These studies also suggest that there is an immune effect from histotripsy therapies across multiple murine tumor types that may be reproducible. Overall, the effects of histotripsy on tumors show a positive effect on immunomodulation.

## Introduction

Although cancer has plagued mankind throughout history, there remains a pressing need to improve treatment options. The core pillars of cancer therapy are chemotherapy, surgery, radiation, immunotherapy, and ablation. The most common treatment option for solid tumors remains surgical resection, even though many patients are not candidates due to tumor size, location, or disease progression (1–4). Tumor ablation modalities have been developed as minimally and non-invasive adjuvants or alternatives to surgery. These procedures include radiofrequency ablation (RFA), microwave ablation, cryoablation, irreversible electroporation (IRE), high-intensity focused ultrasound (HIFU), and histotripsy. While these procedures address many of the issues with traditional surgery, there are still many areas where these modalities can be improved.

While the goal of the field of oncology research is to find curative therapies, novel cancer therapies frequently aim for non-curative endpoints such as increased progression free survival, increased overall survival, decreased tumor burden, and improved quality of life. In many cases, the therapeutic goals of local ablation therapy also include a decrease in tumor burden, enhancement of drug delivery to tumors, and the modulation of the immune system. While immune system modulation offers the most promise in terms of long-term patient benefit, it is currently the least predictable and understood benefit of local tumor ablation therapy. The most observable and reliable benefit of local tumor ablation modalities is currently considered to be the decrease in targeted tumor size (5). This can lead to improved patient outcomes by debulking tumors, which can reduce the patient’s overall tumor burden, improve local vasculature compression, and reduce tumor nutrient utilization.

## The Role Of Ablation In Immunomodulation

**Figure 1:**
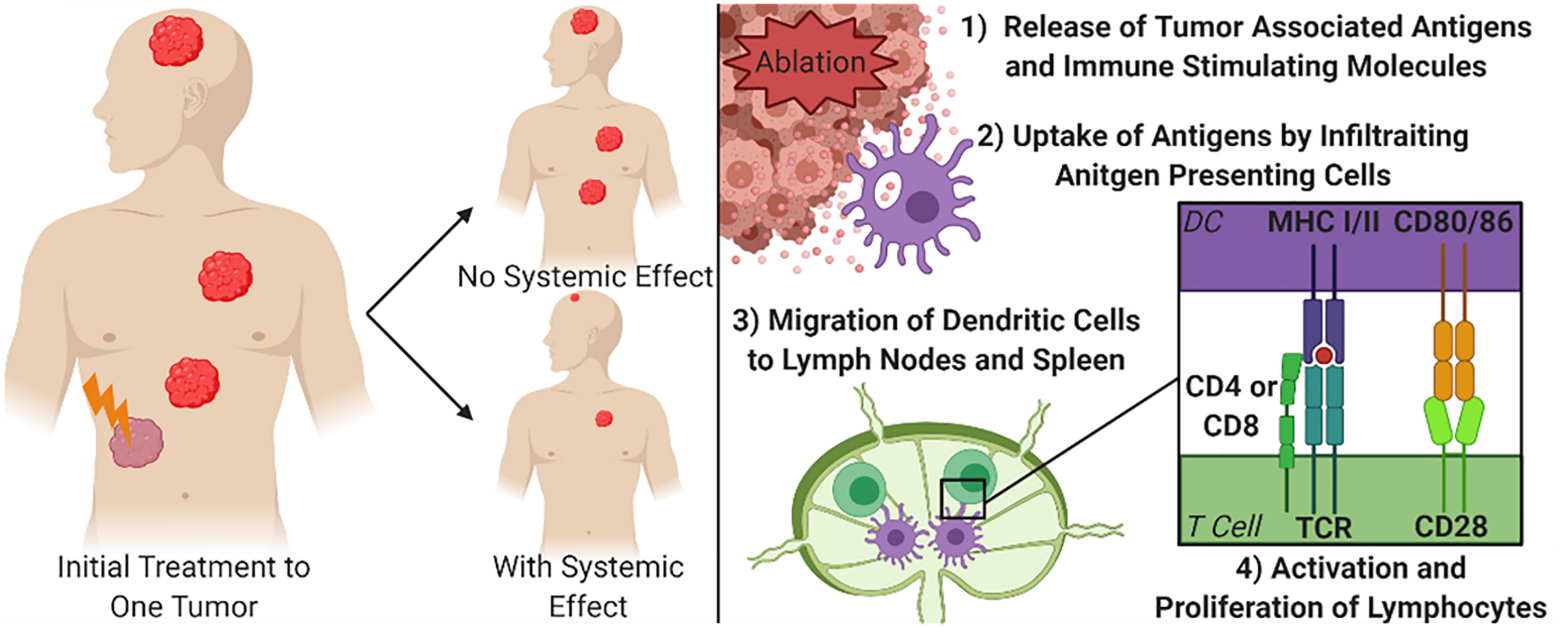
Focal Treatment of Targeted Tumor and Mechanism for Systemic Tumor Control. The right diagram demonstrates the principal of the abscopal effect, where the treatment of one tumor can cause other tumors in the body to shrink or be eliminated to varying degrees due to immune system engagement. The left diagram depicts the simplified mechanism for achieving a systemic effect from focal therapies. DC, Dendritic cell; MHC, Major Histocompatibility Complex; TCR, T cell receptor.

Given that one of the most debilitating aspects of cancer is the burden brought on by metastatic disease, an ideal treatment protocol would address not only debulking larger or debilitating tumors, but also circulating tumor cells and distant metastases. Once cancer has spread from its primary site, focal debulking of individual tumors are less likely to be capable of being a curative treatment option. Therefore, an ideal debulking therapy would not only reduce the size, or fully eliminate, targeted tumors, but would also stimulate the immune system to seek and destroy metastatic lesions throughout the body. This phenomenon has been described as the abscopal effect (**Figure 1**). There is increasing evidence that immune system activation and systemic tumor elimination is critically important for the elimination of micrometastatic lesions distal to the locally treated tumor, that may not be detectable at the time of treatment or surgery. This review briefly introduces the immune response to non-histotripsy tumor ablation therapies in order to provide context and reference to better understand the state of histotripsy knowledge. More extensive reviews of the immune response to non-histotripsy ablation modalities have been previously conducted at depths that are beyond the scope of this review (6, 7).

Before the abscopal effect was formally defined, it was first noted by physicians at the turn of the 20^th^ century who reported that local, targeted radiation of the spleen in leukemia patients would cause changes in the patient’s bone marrow leading to the remission of disease (8, 9). These clinical findings in combination with many mouse studies led to the abscopal effect being defined by the early 1950’s to encompass the effects to distant organs or cancers after applying ionizing radiation (10). This effect was originally thought to only occur for leukemia, where the radiation of the spleen would reduce or eliminate cancerous cells in the body (10, 11). However, over the subsequent decades, additional reports of solid tumors displaying this effect periodically emerged. For instance, the reduction of the sizes of distant metastases were reported after the radiation of a single giant follicular lymphoma or a non-Hodgkin’s lymphoma tumor (12, 13). However, due to the scarcity of reports, the abscopal effect in response to ionizing radiation of solid tumors, including renal cell carcinoma, mammary carcinoma, and neuroblastoma, was frequently dismissed and remained controversial for decades (14–17). To this day, the abscopal effect associated with radiation therapy remains controversial (18). More recently, the development of non-ionizing ablation therapies has led to an increase in the number of abscopal effects reported. Thermal ablations, the first ablation therapies that were developed and widely accepted, also have reported sporadic cases of a systemic immune mediated effect where untreated, distal metastases were found to spontaneously regress (14, 19, 20). One of these earlier case studies showed that the treatment of a primary renal cell carcinoma tumor with RFA caused a spontaneous regression of pulmonary metastases (14). On the other hand, as more non-thermal ablation modalities are being developed pre-clinical studies have had more promising correlations between focal ablation therapy and the systemic effects (21–23). In general, even though there have been promising trends that these therapies can offer systemic therapeutic effects for patients, it should be noted that the reproducibility between patients, tumor types, and therapy modalities is less than optimal (6).

To achieve an abscopal effect, it is not enough to simply release non-specific markers of tissue damage or cell death, if that were the case surgical resections would stimulate systemic effects. Instead, there is a need to release specific tumor associated antigens that allow the immune system to build a targeted response (**Figure 1**). When focused, local ablation therapies break up the stromal tissues of a tumor this can create an increased opportunity for immune cells to physically access the tumor (24, 25). As the cancer cells are ablated, there is an increased abundance accessible tumor antigens that can be recognized by infiltrating antigen presenting cells (APC), such as dendritic cells (20, 26). These APCs can in turn activate lymphocytes to guide a more precise immune response. Locally, activation of cytotoxic CD8+ T cells can increase the clearance of any surviving cancer cells within the area treated with the tumor ablation modality (27). For the strongest systemic immune effects, after ablation, dendritic cells will process the available tumor associated antigens and migrate to the nearest draining lymph nodes and the spleen. Once in the lymphoid tissues, the dendritic cells present the antigens through MHC class I or class II receptors to naïve CD4+ and CD8+ T cells, respectively, along with co-stimulation of CD28 with CD80/86. Studies that show an increase in tumor specific lymphocytes in secondary lymphoid organs or in peripheral blood have also established the correlation between these activated cells and systemic decreases in tumor burden (28–31). More recently, additional studies have also defined B cell activation, the maintenance of plasma cells, and even the generation of tumor specific antibodies following local tumor ablation and dendritic cell activation (29, 31). For example, a study treating hepatocellular carcinoma with RFA reported that only 91 of the 178 patients that had a decreased neutrophil-to-lymphocyte ratio, which is an often used biomarker of systemic immune system activation, independently correlated to a significantly increased survival when compared to patients that did not see the increased ratio (32). While the activation of T and B lymphocytes is a core feature of the systemic effects of local tumor ablation therapy, there are many inconsistencies. Similarly, in a prospective study investigating the long-term effects of RFA after treating secondary liver tumors, it was found that only 6 of 49 patients studied had increased antibodies, CD4+ T cells, and/or CD8+ T cells months after treatment (29). While the exact mechanisms underlying the inconsistent reports of the abscopal effect and systemic anti-tumor immune responses are yet to be fully defined, changes in the local tumor microenvironment have been postulated to significantly impact treatment success.

Although there any many promising pre-clinical and clinical studies showing that ablation therapies have positive effects on systemic immune response, there have also been reports of the opposite. Hepatic RFA and microwave ablation have both seen pre-clinical reports of the treatment of hepatocellular carcinoma tumors and colorectal metastases stimulating growth of existing and new tumors (33, 34). There has been multiple hypothesis investigated to explain this phenomenon. One main hypothesis to explain this phenomenon focuses on the sub-ablative hyperthermia that effects margin zone tissues. Studies investigating this effect have shown upregulation of inflammatory pathways in this region, including the IL-6-HGF/c-Met-STAT3-VEGF axis and the HSP70 related pathways (35–37). One of these studies also demonstrated that this systemic effect is minimized when the RFA is applied to the healthy liver of rats baring mammary adenocarcinoma tumors at a higher energy dose over a shorter time in order to reduce the damage to surrounding tissues and when administered in combination with HSP inhibitors (35). Another cause associated with increased tumor growth after the application of a minimally invasive ablation, unrelated to changes in the inflammatory response, is the seeding that can occur along the needle track (38).

### The Tumor Microenvironment and Tumor Associated Immune Cells

To determine the cause of systemic anti-tumor immunity, and therefore how to modulate it, there is a need to understand what is occurring within the tumor microenvironment. Within the heterogeneous cell populations of most tumors, there are a variety of tumor-associated immune cells. In an ideal case, these leukocytes are eliminating malignant cells. However, in the case of many cancers, the leukocytes are reprogrammed, or polarized, to support tumor escape and function to promote tumorigenesis through the generation of a relatively immunosuppressive tumor microenvironment. These tumors are often referred to as being immunosuppressive, or immunologically “cold,” due to their lack of inflammation and active immune suppression. With multiple types of pro-tumor immune cells that produce factors that can improve the tumor niche, it is difficult for the pro-inflammatory/anti-tumor immune response to overcome this “cold” environment.

**Figure 2:**
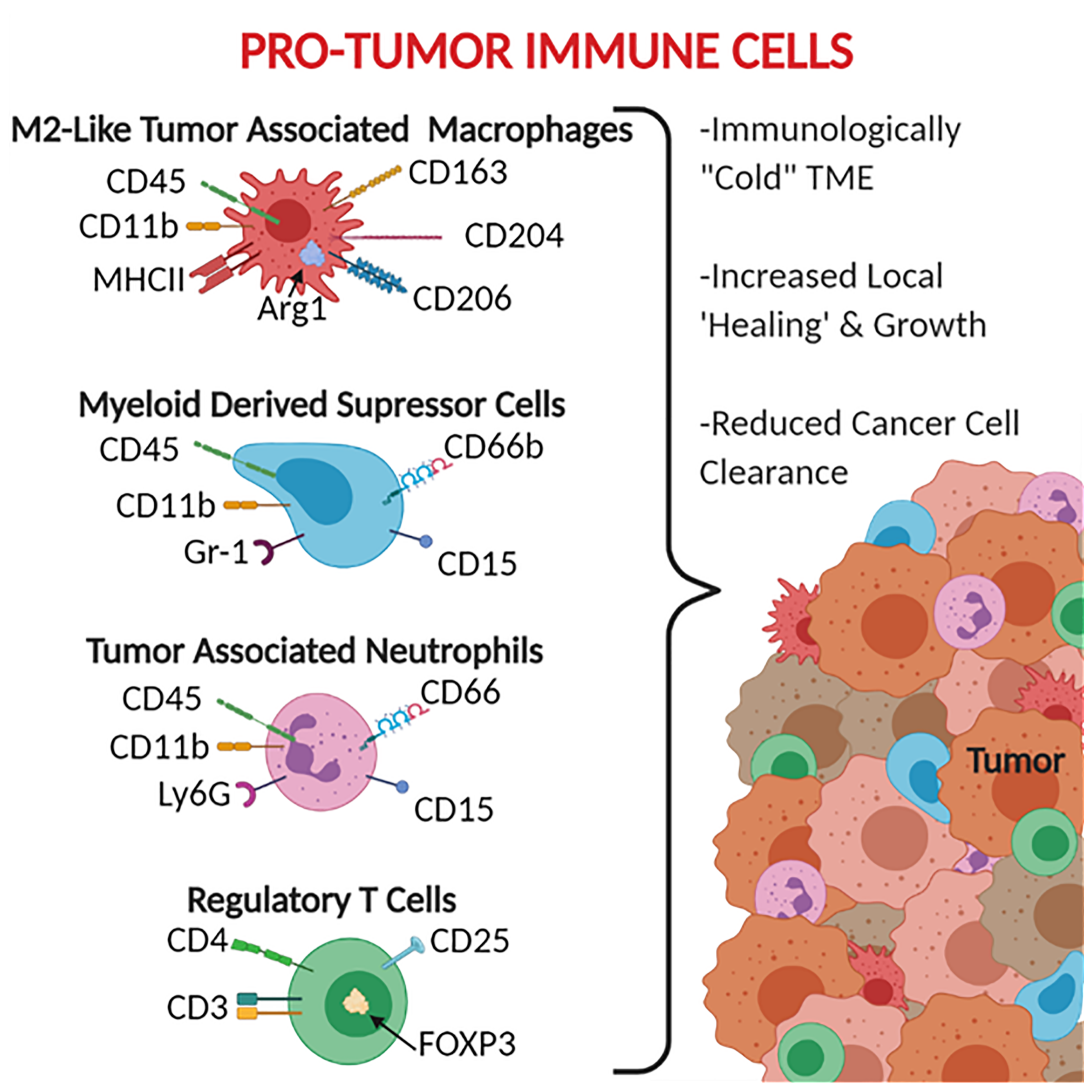
Pro-Tumor Immune Cells. CD66, CD66b, and CD15 are human specific markers. Gr-1 represents both Ly6C and Ly6G, therefore does not distinguish the MDSC subtypes, MDSCs are monocytic when Ly6C+ and granulocytic when Ly6G+. Most of the markers available for pro-tumor immune cells are surface receptors and ligands, exceptions listed here include the intracellular protein Arg1 and the transcription factor FOXP3.

The leukocytes most commonly associated with these anti-inflammatory and tumor promoting properties include tumor-associated macrophages (TAM), myeloid-derived suppressor cells (MDSC), and tumor-associated neutrophils (TAN) (**Figure 2**). TAMs are generally defined as CD45+ Ly6C^-^ MHCII^+^ CD11b^+^ (39, 40). TAMs are also broken down into subsets based on polarization to the M1 macrophage-like phenotype (pro-inflammatory/anti-tumor) CD68^+^ CD80/86^+^ CD11c^+^ iNOS^+^ and the M2 macrophage-like phenotype (pro-tumor) CD163^+^ CD204^+^ CD206^+^ Arg1^+^, with M2-like TAMs being the most immunosuppressive (41, 42). It should be noted that while the M1-like and M2-like TAMs do share similar expression patterns and activities, they are not the same as the traditional M1 and M2 polarized macrophages. MDSCs are generally defined as CD45^+^ CD11b^+^ CD11c^-^ MHCII^-^ as well as CD15^+^ and CD66b^+^ in humans (43–45). MDSCs are broken into two main subtypes: monocytic MDSCs Ly6C^+^ Ly6G^-^ and granulocytic MDSCs Ly6C^-^ Ly6G^+^ (43). When generally searching for MDSCs, the GR-1 antibody has been used given that it binds to both Ly6C and Ly6G (43). TANs are defined as CD45^+^ CD11b^+^ Ly6G^+^ and have been reported to express CD66 and CD15 in human cancers (46–48). It should be noted that the markers that have been established for TANs are indistinguishable from traditional neutrophils, with the most established phenotypic difference between the two cell groups being that neutrophils have a half-life of 6-8 hours whereas TANs have a significantly increased lifespan (an additional 12-24 hours) caused by an inhibition of apoptotic pathways and support from cytokines within the tumor microenvironment (48–52). Similar in nomenclature to TAMs, TANs can also be divided into N1 pro-inflammatory and N2 pro-tumor subtypes, however due to limitations in markers there is not an established panel for differentiating N1 from N2 TANs (46).

These anti-inflammatory leukocytes are typically distributed throughout the tumor and margins (**Figure 2**) and function beyond simply being tumor associated. These leukocytes are frequently referred to as being pro-tumor given that the range of their activities can actively promote tumor growth and development. These functions include reducing the impact of anti-tumor immune responses, recruiting additional tumor supporting cells, helping the tumor grow by stimulating angiogenesis and secreting growth factors, and assisting in the epithelial-to-mesenchymal transition (53–55). For TAMs, this includes producing anti-inflammatory mediators such as IL-10, Arg1, and TGF-β (56–58), promoting the expression of checkpoint inhibitors to suppress T cells (56, 57), inducing cancer stem cell proliferation *via* IL-6 signaling through STAT3 (59), and producing VEGF to stimulate angiogenesis (60, 61). These effects are also seen in TANs and MDSCs, which have both been shown to produce similar profiles of cytokines, checkpoint inhibitors, and growth factors (47, 48, 62–64).

In addition to these monocyte and granulocyte derived cells, lymphocytes can also aid in maintaining this “cold” tumor microenvironment. Regulatory T cells, defined as CD3^+^ CD4^+^ CD25^+^ FOXP3^+^, can suppress the adaptive immune response (65). While MDSCs, TANs, and TAMs are commonly identified as tumor promoting cells with specific markers and functions, the role of T regulatory cells is more situationally specific, and their presence and ratio are not necessarily a sign of an immunosuppressive tumor microenvironment. In most cases, the presence of a high ratio of T regulatory cells, known for inhibiting the function of other T cells, is a sign of an immunosuppressive tumor microenvironment. Clinically, in ovarian carcinoma it has been established that the recruitment of T regulatory cells into the tumor stroma leads to decreased survival of patients (66). Additionally, for pancreatic cancer, the T regulatory cells have been found to aid in the pro-carcinogenic inflammation driven by T helper 17 cells (67). These poor prognostic correlations in patients is tied to the fact that T regulatory cells can suppress the function of T helper cells through the production of immunosuppressive cytokines, including IL-10 and TGF-β (68, 69), secrete perforins and granzymes to directly destroy effector T cells and B cells (70), and express the checkpoint inhibitor CTLA-4 to suppress APC activity (71). There have also been reports of T regulatory cells directly increasing the growth of tumors through the stimulation of cancer stem cells *via* the NF-κB-IL6-STAT4 signaling axis (72).

On the other hand, there are reports of certain cancers, such as colorectal cancer, which have worse prognoses for patients that have a reduced presence of T regulatory cells within their tumors (73, 74). A proposed mechanism for the seemingly contradictory immune promoting function of T regulatory cells in colorectal cancer is associated with the location of these tumors. In the gastrointestinal tract, T regulatory cells are primed against the microbiota of the intestinal space instead of the cancer (74). This secondary, non-tumoral target for the immune system allows for the immune response to become more robust in the colon, which can in turn destroy the cancerous cells, without being hampered by the immunosuppressive control of the tumor microenvironment.

### Mechanisms for Shifting the Tumor Microenvironment to Pro-Inflammatory

**Figure 3:**
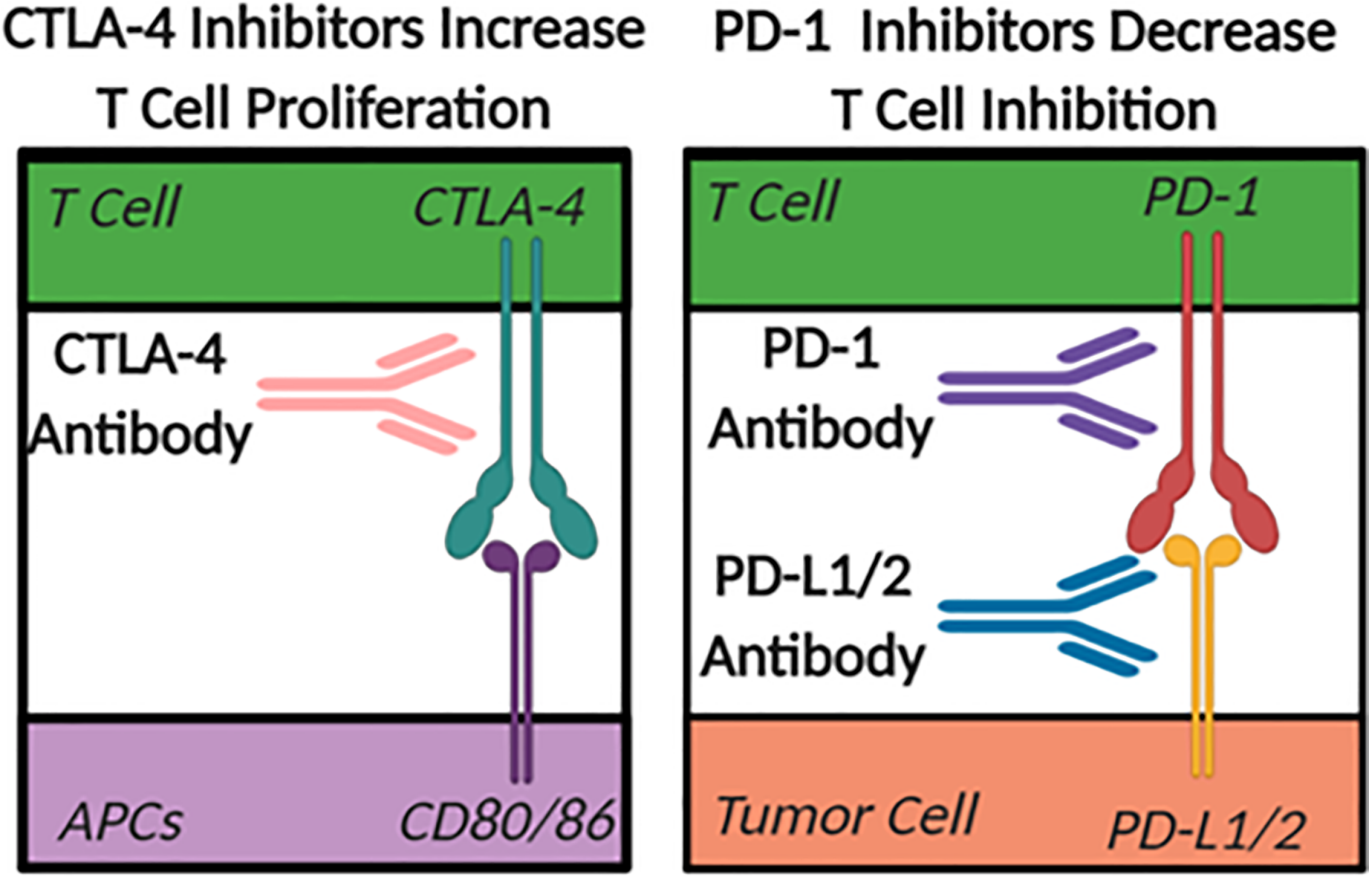
The Role of Check Point Inhibitors. Antigen Presenting Cells (APC).

The largest difference between a “cold” and a “hot” tumor microenvironment is typically considered to be related to the infiltration of anti-tumor, pro-inflammatory T cells in “hot” or inflamed tumors (75). Checkpoint inhibitors are emerging as the primary therapeutics used to facilitate the shift in the tumor microenvironment from “cold” to “hot” or immunologically active (76). In the case of many cancers, the checkpoint inhibiting pathways are hijacked to prevent immune cells from eliminating unhealthy or cancerous cells. The two most heavily studied checkpoint inhibitor pathways in the tumor ablation field are CTLA-4 to CD80/86, blocking the normal co-stimulatory role of CD80/86 in T cell activation, and PD-1 to PDL1/2 (**Figure 3**). CTLA-4 is a checkpoint inhibitor most commonly associated with antigen presentation in the lymph nodes that minimizes the proliferation of T cells (76). Therapeutically, when CTLA-4 on T cells is blocked with a targeted antibody, there is an increase in overall T cell proliferation and an increased probability of forming a tumor-specific immune response. While anti-CTLA-4 is used to increase T cell proliferation, PD-1 inhibitors decrease T cell inhibition and improve function. Under normal conditions, PD-1 inhibits the activity of T cells and prevents the targeting of healthy host cells by T lymphocytes (76). This pathway in healthy tissues prevents overzealous immune responses that can drive autoimmunity. However, some tumor cells hijack this mechanism and increase the expression of the PD-1 ligands (PD-L1/2), which artificially reduce the activity of anti-tumor T cells within the tumor microenvironment. The use of antibodies targeting the PD-1 pathway for cancer therapy has shown increased levels of infiltrating T cells in tumors that express the PD-L1/2 surface receptors. While PD-1 pathway inhibition has a more tumor-specific therapeutic effect, and typically fewer side effects compared to CTLA-4 based therapies, there is still a need to improve the implementation of checkpoint inhibition strategies. This is especially true in immunologically “cold” tumors that achieved immunosuppression without the use of PD-1 or CTLA-4 pathways where these checkpoint targeted therapeutics have proven to be minimally effective. Unfortunately, even in cases where these therapies work there are often significant side effects. For example, the systemic increase in T cell abundance often leads to autoimmune disease-like symptoms such as fatigue, nausea, vomiting, diarrhea, arthritis, dermatitis, and myalgia (77).

Beyond the biochemical changes in the tumor microenvironment that work to subvert immune system detection and elimination, physical barriers that can have significant detrimental effects on therapeutic approaches are also present. Indeed, many tumors adopt organ-like structures and extensive fibrosis that build highly complex physical barriers (78, 79). Given that the checkpoint inhibitor’s mechanism is to improve immune system activation and targeted killing of tumor cells, it is critical that leukocytes have physical access to the malignant cells. In an attempt aid the checkpoint inhibitor therapies, ablation modalities have been extensively studied to better understand their ability to modulate the immunological aspects of the tumor microenvironment. While debulking the tumor and reducing a patient’s overall tumor burden is the primary goal of focal tumor ablation therapies, improved immune system access is proving to be an added benefit that can shift the local TME from being “cold” and immunosuppressed to more “hot” and immunostimulated.

Ablation therapies with a thermal effect have sporadically seen pro-inflammatory changes in the tumor microenvironment in the weeks and months following treatment (80–83). However, in terms of immunomodulatory success, non-thermal modalities seem to have more reproducibility, predictability, and improved pre-clinical and clinical success (21, 84–86). The leading hypothesis regarding differences between modalities theorizes that while both thermal and non-thermal ablation approaches kill tumor cells, non-thermal ablation modalities release higher levels of native tumor-specific antigens that have not been distorted or denatured by heat. For example, one *in vitro* study showed that intact neoantigens are released in significantly higher magnitudes from cells treated with cryoablation and IRE compared to thermal ablation. Not only were there more tumor antigens released, these antigens were also more potent at stimulating dendritic cell antigen presentation and cytotoxic CD8+ T cell activation (87). Activated lymphocytes, either in response to tumor-specific antigens or more generally in response to innate immune system activation driven by the increase in damage-associated molecular patterns (DAMPs) further promote the shift from a “cold” to a “hot” tumor microenvironment (21, 88, 89). Thus, it is becoming clear that non-thermal ablation modalities are capable of inducing robust local and systemic anti-tumor responses, as either an independent or an adjuvant therapy with immunotherapeutics, to offer significant promise beyond just focal tumor debulking.

## Histotripsy: Non-Thermal, Non-Invasive Tumor Ablation

**Figure 4:**
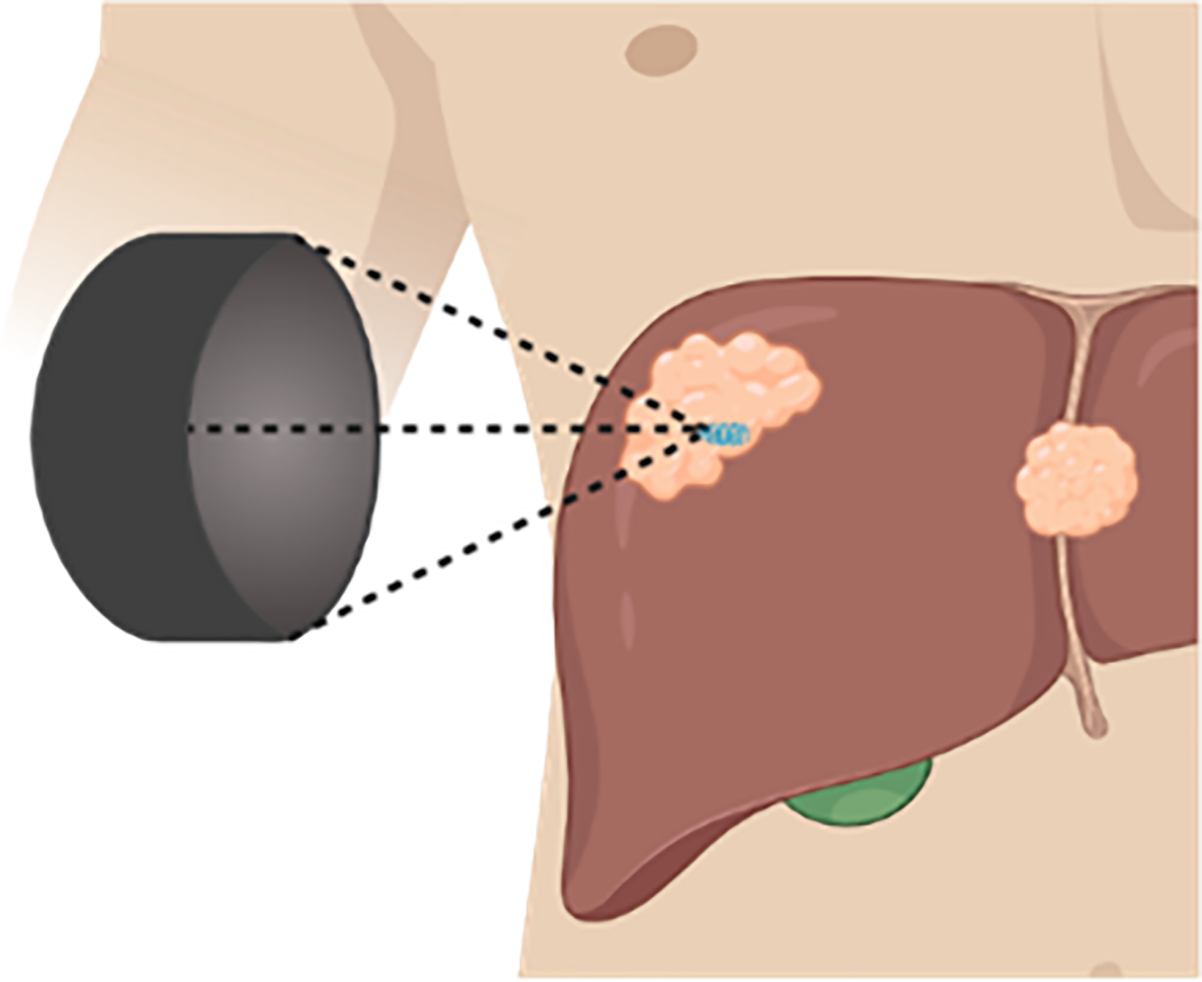
Histotripsy Schematic. Therapeutic ultrasound transducer positioned outside of the body, focuses ultrasound waves within a targeted tissue generating a cavitation bubble cloud.

Histotripsy is a non-thermal focused ultrasound therapy that utilizes microsecond (cavitation-cloud histotripsy, CCH) or millisecond (boiling histotripsy, BH) pulsing regimens to generate cavitation bubble clouds that leads to precise non-thermal tumor ablation (**Figure 4**) (90). In addition to histotripsy, other non-thermal focused ultrasound methods that induce cavitation and do not induce substantial thermal effects are broadly referred to as mechanical HIFU (mHIFU), as opposed to conventional HIFU that typically refers to thermal ablation. For the purposes of this study, thermal HIFU will be referred to as tHIFU to delineate from mHIFU procedures, which will be the focus of this review.

In histotripsy, the rapid expansion and collapse of cavitation bubbles ablate tissues into acellular debris (91–94). This non-thermal ablation process is well-established and has resulted in important hallmarks of histotripsy, including high precision and tissue selectivity (95–97). For example, tissues with a higher Young’s Modulus, such as vasculature and collecting ducts, are more resistant to damage (98–105). Unlike the vast majority of other ablation therapies, because histotripsy is non-thermal, it is not affected by the heat-sink effect, and therefore remains safe and efficacious for use near the vasculature (106, 107). Additional benefits of histotripsy include real-time imaging feedback with standard imaging (ultrasound, MRI, CT), highly precise-millimeter accuracy, and the ability to treat tumors of arbitrary sizes and shapes (100, 108). After treatment, tissues treated with histotripsy have shown more rapid dissolution of the ablated tissues compared to other ablation modalities (92, 108). For instance, CCH ablation of healthy rat livers showed rapid involution of treated volumes, granulation, and growth of healthy hepatocytes within 28 days with minimal scarring (92). This healing process is more rapid than what has been reported for other ablation modalities, such as RFA in humans, which has been reported as causing “thermal fixation,” where the necrotic mass is still present 14 months post-treatment due to thermal denaturation of the tissue leaving it resistant to being broken down by normal pathways (109).

**Figure 5:**
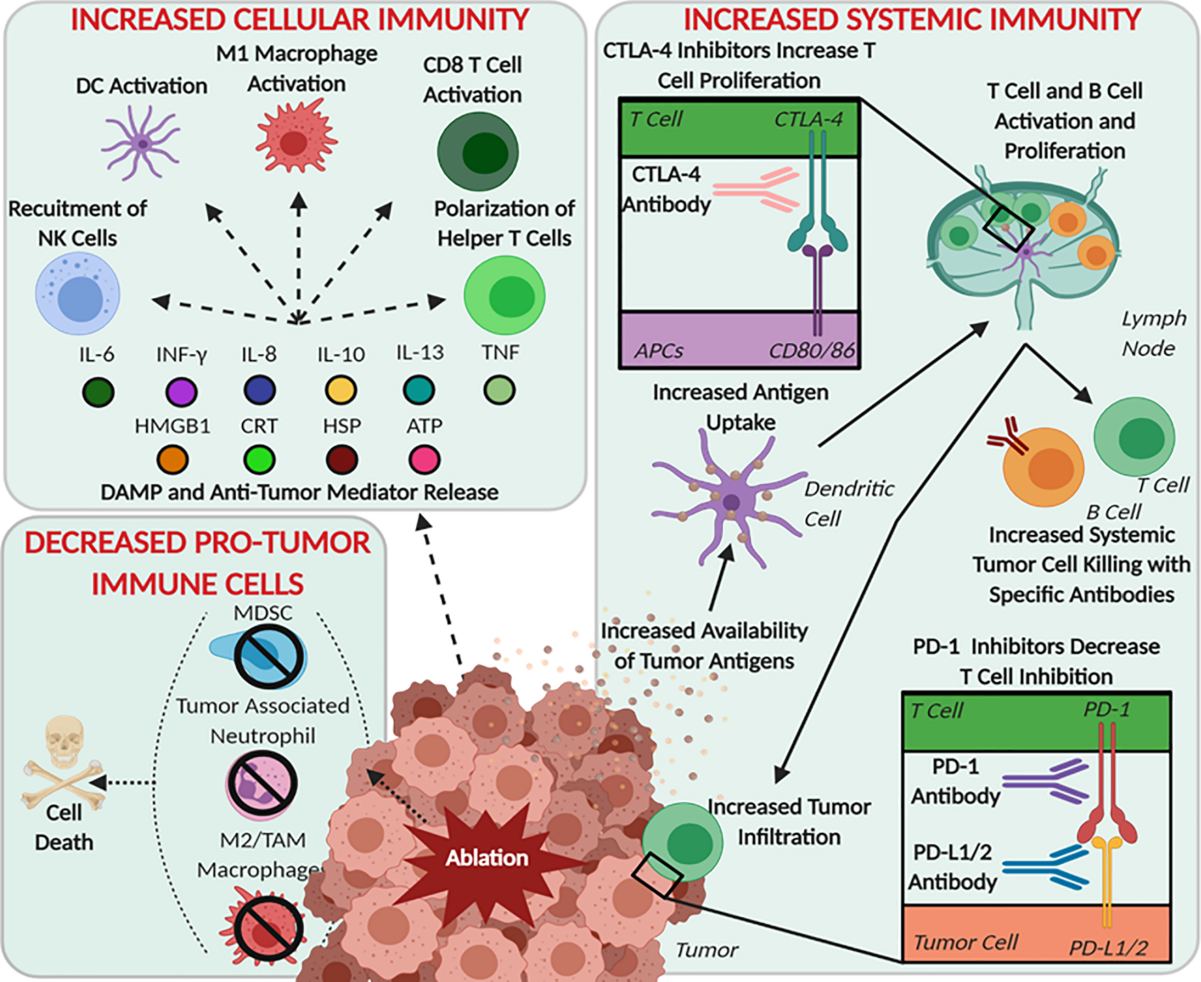
Schematic of Histotripsy’s Postulated Roles in Immune System Modulation. Immune processes, cells, and molecules that have been shown to be modulated by focused ultrasound therapies after mechanical ablation are summarized based upon immune system functions: pro-tumor immune cells, cellular immunity, and systemic immunity.

In mouse models, histotripsy ablation of hepatic, renal, neuroblastoma, melanoma, and pancreatic tumors has resulted in significantly increased survival (22, 110–113). In addition to debulking tumors, recent evidence has suggested that histotripsy has the capability to induce a systemic immune response, as evidenced by the attenuation of metastasis and an improvement in local and distant disease with combination immunotherapy (22, 114, 115). This effect has been shown in single tumor treatments and has also shown abscopal-like decreases in contralateral tumor growth in a separate untreated tumor (22, 112, 115). This effect was found to be modest, but statistically significant with histotripsy ablation compared to the insignificant trend in mice treated with irradiation and radiofrequency ablation (22). The molecular immunological effects caused by histotripsy, independently and compared to other ablation modalities, can best be summarized by the proposed mechanism shown in **Figure 5** that is described in the sections below.

## Immunologic Effects Of Histotripsy

The immunological responses to histotripsy ablation through cavitation, regardless of the sub-therapy being applied, are theoretically similar due to comparable effects on targeted tissues. Cavitation breaks down tissues, ablating cells into subcellular fragments and acellular debris (116). To date, there have been no studies to suggest that mHIFU, BH, nor CCH have any significant differences in immunologic responses. Therefore, for this review, the immune responses to these modalities will be reviewed together.

### Decreases in Pro-Tumor Immune Cells

Being able to reduce the magnitude of tumor supporting immune cells present within the tumor microenvironment is pertinent due to the correlation of these cells with poorer outcomes (54, 117, 118). There have been multiple studies on thermal and non-thermal ablation modalities that show either the therapy directly eliminates cells that support the tumor microenvironment or the damage initiated by the ablation reprograms the leukocytes within the tumor microenvironment to shift the microenvironment to one that is more harsh towards tumor progression (6, 7).

In the case of histotripsy, there has been one report from Pahk et al. demonstrating the supernatant from cells treated with BH *in vitro* polarizes naïve macrophages (M0) into anti-tumor macrophages (M1) and the redifferentiation of tumor associated macrophages (M2-like phenotype) to the more inflammatory M1 phenotype (119). In this study, breast cancer cells were treated with BH at varying doses *in vitro* and the supernatant collected. Analysis found increased pro-inflammatory signaling molecules including TNF, which is a potent and well established M1 stimulating cytokine (120, 121). After THP-1 human monocyte cells were cultured with the BH supernatant they displayed physical and morphological changes consistent with polarization to M1 macrophages and showed significantly elevated gene expression consistent with M1 polarization (119). Together, the data presented in this study suggest that monocytes and macrophages near the histotripsy ablation region polarize to pro-inflammatory, anti-tumor phenotypes. Likewise, Pahk et al. continued *in vitro* studies showing that the BH supernatant can also stimulate M2 cells to repolarize to M1, thus leading to a decrease in M2 pro-tumor macrophages (**Figure 5**). Thus, these data further suggest that any tumor-associated macrophages remaining within the treatment zone have the potential to repolarize from an M2-like state to M1, similar to the cells at the margins of treatment. If this can be recapitulated *in vivo*, this would help further decrease the presence of pro-tumor immune cells and could significantly alter the shift the tumor microenvironment from “cold” to “hot.”

Regarding *in vivo* studies, it may seem safe to assume that pro-tumor cells within the tumor microenvironment of the treatment zone would be ablated due to subcellular fractionation of histotripsy. This specific hypothesis is not confirmed by literature, and there is still a window for any cells that survive partial ablation to proliferate and maintain the pro-tumor tumor microenvironment. However, this hypothesis is consistent with results following RFA, microwave ablation, and IRE, which can all reduce the presence of tumor supporting immune cells in the tumor microenvironment (7, 122). This dynamic has also been seen in human patients. For instance, studies of pancreatic adenocarcinoma patients treated with IRE found a decrease in T regulatory cells in peripheral blood in the days following treatment (85, 86). Another study of patients with primary liver tumors treated with RFA showed a strong correlation between the decrease in MDSCs and the increase in patients’ survival (123). The importance of the attenuation of pro-tumor immune cells, their decrease in function, and reduction in magnitude after ablation are all generally correlated with reduced survival in human patients. Therefore, it is important for the field of histotripsy to further understand the effects of the ablation on this aspect of the immunomodulatory mechanism.

### Histotripsy Produces Damage Associated Molecular Patterns That Directly Increase Local Cellular Immune Responses

Histotripsy generates subcellular fragments of targeted cells through mechanical fractionation (116). This, in turn, releases a multitude of damage associated molecular patterns (DAMPs) that have the potential to stimulate the innate immune system and significantly alter the tumor microenvironment. DAMPs are molecules that are found extracellularly following most forms of pathological cell death. These molecules are readily identified by the immune system, which in turn elicits a robust innate immune response. Several DAMPs have been observed and evaluated in the context of tumor ablation, including the endoplasmic reticulum associated protein calreticulin (CRT), the non-histone nuclear binding protein HMGB1, the intracellular energy molecule adenosine 5’-triphosphate (ATP), and various heat shock proteins (HSP) that were originally identified as DAMPs released after exposure to elevated temperatures (124–128). Once released from the injured cells or neighboring cells following damage, these molecules can stimulate a robust immune response by binding to receptors known as pattern recognition receptors. As an example, HMGB1 commonly activates the Toll-like Receptors 2 and 4, which classically signals through NF-κB to drive multiple biological responses, including inflammation and cell death (129).

These DAMPs are emerging as key mediators of the ensuing immune response following histotripsy. Specifically, following histotripsy treatment of cancer spheroids, HMGB1, CRT, and HSP were identified in cell supernatants and correlated to levels expected from within the tumor after an *in vivo* ablation (119). Additionally, murine tumors treated with histotripsy have been found to have elevated levels of HMGB1, CRT, and HSP within the tumor and increased HMGB1 has been routinely found in serum following treatment (22, 110, 130). DAMPs released in serum function to systemically prime the immune system and can function to recruit increased circulating leukocytes that can ultimately congregate at the site of tissue injury following ablation. Together, the local and systemic presence of DAMPs aid additional immune cell migration, shift the tumor microenvironment, and may eventually serve as effective biomarkers of ablation success.

While DAMP release has been confirmed following histotripsy, it is also critical to determine if these signals are in their naïve and unaltered structural configurations. For thermal ablation modalities, it has been well established that proteins can be denatured under high heat settings, making them less efficacious and predictable in stimulating the immune system. One study investigated the difference in the release of ATP and hsp60 between mHIFU and tHIFU (130). This study found that mHIFU is capable of releasing higher levels of both molecules after treatment, and that the DAMPs released from mHIFU were more capable of stimulating downstream immunologic changes, such as activating dendritic cells (130). The ability of mHIFU to release a higher magnitude of active DAMPs compared to tHIFU adds to the hypothesis that non-thermal ablation therapies are more immunomodulatory compared to thermal modalities.

These data point to histotripsy therapies having the ability to release molecules that can drive anti-tumor immune system activation, for example by initiating a robust innate immune response. However, the data associated with the specific DAMPs released following histotripsy is not consistent across studies. For example, a recent study of subcutaneous murine neuroblastoma treated with mHIFU showed no significant changes in HMGB1 levels (112). This could be due to a difference in tumor types. For example, neuroblastoma did not see the change in DAMPs after histotripsy that was observed in colon adenocarcinoma, renal cell carcinoma, and breast adenocarcinoma reported elsewhere (22, 110, 112, 130). Alternatively, there are potential differences in histotripsy and other mHIFU ablation modalities that are not fully understood and may have played a role. Another possibility is that differences in DAMP release between a partial ablation versus a more complete treatment are responsible for these differences. All of these variables could potentially affect the quality and quantity of DAMPs released. Likewise, the actual DAMPs generated could significantly vary depending on a number of experimental factors. Although the aforementioned DAMPs are currently the most defined following histotripsy, any DAMP could conceivably function as an activator of the innate immune system after ablation. Future investigation into the effects of other DAMPs, such as nuclear and mitochondrial DNA, reactive oxygen species, and calcium ions could prove useful for improving adjuvant therapies (131). Additional studies are needed to determine any potential difference in the quantity and quality of the release of DAMPs from the different mechanical focused ultrasound therapies.

### Pro-Inflammatory Cytokines and Chemokines Associated With Histotripsy Based Tumor Ablation Modalities

Cytokines and chemokines are critical for cell-to-cell communication and immune system modulation following damage (**Figure 5**). These molecules stimulate the differentiation and activation of local immune cells and systemically recruit additional cells to the site of damage. One of the critical cytokines found to be significantly altered in multiple histotripsy studies is IFNγ (22, 112, 115, 119). In an *in vitro* study on breast adenocarcinoma and an *in vivo* murine study of neuroblastoma, there was an approximately 2-fold increase in IFNγ after histotripsy (112, 119). However, in an *in vivo* study where Eker rat renal cell carcinoma tumors were treated with histotripsy, there was a significant decrease, ~6pg/mL down to ~2pg/mL, in treated kidneys (115). This decrease was attributed to the native kidney tissues’ defense mechanism to protect against acute and chronic kidney injuries (132). The importance of changes to IFNγ is highlighted by its role in activating anti-tumor APCs and effector cells, inducing ischemia within the tumor by acting on the endothelial cells thus reducing the stability of intratumoral vasculature, and initiating tumor cell death through the activation of apoptosis and necroptosis pathways (133). However, IFNγ is also a double-edged sword in the tumor microenvironment. In addition to its function as a potent pro-inflammatory mediator, IFNγ can also up-regulate PD-L1 in some tumors as part of the IRF1 axis, resulting in immune evasion (134).

While IFNγ is the most consistently reported inflammatory mediator across therapies and tumor types, other important cytokines have been reported as either significantly or notably upregulated in the days after histotripsy treatment including IL-6, IL-2, TNF, IL-8, IL-13, and IL-10 (22, 110, 112, 119). While more research is needed to more accurately determine the state of cytokines in specific tumor types after mechanical ablation, the general picture appears positive. However, high levels of IL-6, IL-10, and IL-8 in serum have been reported to negatively correlate with survival in pancreatic cancer patients, due to their role in stimulating inflammation and adverse changes in the tumor microenvironment that spurs the growth and development of pancreatic tumors (135, 136). Although this could correlate to a foreboding warning against histotripsy therapies, at least for pancreatic applications, the induction of these pathways actually correlates with improved anti-tumor immune responses and increased survival in mice (22). It is certainly possible that these results simply reflect the commonly promoted differences between mice and humans. However, it is also possible that additional mechanistic insight may be necessary to better translate the findings from rodents to human patients.

In addition to the cytokines released there are changes in the serum levels of multiple growth factors as well. For example, after mHIFU ablation of neuroblastomas, both an increase in GM-CSF and a decrease in VEGF were found within 24 hours of treatment (112). Increased levels of GM-CSF are positively correlated with increased APC cell differentiation and activation. Decreased levels of VEGF are positively correlated with better clinical outcomes, due to the rate of vascularization of tumors affecting tumor growth and immunosuppressive effects (137). The finding that histotripsy can increase GM-CSF and decrease VEGF adds to the potential for histotripsy to shift the tumor promoting immune microenvironment to one that is more proinflammatory and tumor suppressive.

### Histotripsy Significantly Alters Immune Cell Populations Systemically and in the Tumor Microenvironment

With the increased release of DAMPs and anti-tumor mediators, there is a resultant change in intra-tumoral immune cell populations. In response to damage, the cells associated with innate immunity are the most rapidly recruited. For histotripsy ablation, this has been reported to include neutrophils, natural killer cells, dendritic cells, and macrophages (**Figure 5**). In an *in vitro* experiment, the supernatant of mHIFU treated murine colon adenocarcinoma cells was shown to activate macrophages (130). This macrophage activation was established through the release of TNF from the macrophages, which was significantly greater after mHIFU compared to both tHIFU and controls (130). Additionally, *in vivo* treatment of melanoma tumors with CCH showed increases in neutrophils, natural killer cells, dendritic cells, and macrophages 10 days after treatment (22). This increase in intratumoral immune cell populations indicate that the tumor microenvironment has become more immunostimulatory and these cell populations typically correlate to pro-inflammatory and anti-tumor changes in the tumor microenvironment.

Beyond innate immune cells, modulation of adaptive immune cells after ablation has also been strongly correlated with clinical success (6, 7). In the same melanoma CCH ablation study, increases in T helper cells and B cells within the treated tumors were observed 10 days post-treatment (22). In addition to the increase in these adaptive immune cell populations, the study also found a decrease in intratumoral T regulatory cells (22). The increase in helper T cells and B cells is a further indication that the TME has shifted to a more permissible, pro-inflammatory environment and further illustrates the improved accessibility of these cells to the tumor. In general, the influx of leukocytes appears to reflect the early induction of the innate immune system and eventually yields to cells associated with a robust adaptive immune response over the course of a few weeks following treatment. Together, these changes are predicted to have significant impacts on the local and systemic effects on metastatic lesions. However, it is critical to note that, while the increased presence of these pro-inflammatory immune cells within the residual treated tumor does hint at a stronger and more robust overall immune response, these cells must be specifically primed for tumor antigens found and either in the circulation or in secondary lymphoid organs to effectively control the overall disease burden.

### Improved Engagement of the Adaptive Immune System and Increases in the Systemic Anti-Tumor Immune Response

The initiation of the systemic anti-tumor immune response requires the generation of both high quantity and high-quality tumor antigens. Previous studies with thermal ablation, cryoablation, and IRE established that the non-thermal ablation modalities appear to release antigens that are significantly better at driving predictable and effective antigen presentation (87). This ultimately results in improved systemic immune responses (87). Although an explicit study has yet to explore if histotripsy ablation negatively impacts the quality of released antigens, studies have been conducted that indirectly evaluate this mechanism. A study using the B16F10 murine melanoma cell line and a transgenic variant of the cell line revealed an increase in the immunomodulatory effects of histotripsy in the tumors that expressed an immunogenically active antigen (22). In this study, only mice that had tumors transfected with the potential antigen GP33, a known antigen from lymphocytic choriomeningitis virus, and were also treated with histotripsy, generated CD8+ T cells that were capable of producing IFNγ after stimulation with IL-2 and brefeldin A. These two agents stimulate memory CD8+ T cells. Together, these data imply that transfected antigens are still viable after histotripsy treatment and are able to stimulate an adaptive immune response.

The activation and migration of dendritic cells to tumor-draining lymph nodes and the spleen are further evidence of systemic immune system activation. Early studies into dendritic cells found the supernatants collected from *in vitro* mHIFU treatments were able to stimulate immature dendritic cells to express higher levels of CD80 and CD86, which are markers of activation, and enhanced secretion of IL-12, which are utilized by dendritic cells to activate both CD8+ cytotoxic and CD4+ helper T cells (130, 138, 139). Additional murine studies with various tumor types further established the migration of, or the increase in, the number of dendritic cells to the tumor-draining lymph nodes and spleen (112, 140, 141). The higher presence of this critical antigen-presenting cell indicates that a subsequent increase in lymphocyte populations is likely.

Once the APCs have been activated, antigens are presented CD8+ and CD4+ T cells that proliferate and are subsequently recruited back to the treated tumor, as well as, circulate systemically to target and kill distal tumor cells expressing the targeted tumor specific antigens. As discussed earlier, the reduction of T regulatory cells is important for changing the tumor microenvironment and shifting the immune response from “cold” to “hot”, allowing these anti-tumor CD4+ and CD8+ T cells access to malignant cells. Histotripsy has been shown to reduce the magnitude of T regulatory cells and increase the ratio of CD8+ to T regulatory cells in both the tumor-draining lymph nodes and spleens of treated mice (22, 141). For example, the ratio of CD8+:CD4+ cells in the spleen was found to increase after treating melanoma tumors with CCH (22). Additionally, after treating prostate tumors with mHIFU, the ratio of CD8+:CD4+ cells in the spleen was also found to be increased, and protective against subsequent tumor challenges (141). However, in treating neuroblastoma tumors with mHIFU, the ratio of CD8+:CD4+ cells in the spleen was found to decrease (112). This difference in the increase versus decrease of the CD8+:CD4+ ratio is not necessarily a sign that regulatory T cells are predominate nor is it indication of poor prognosis. This shift in ratio could indicate helper T cells primed to increase an immune response in response to the damage that could lead to an immunostimulatory response. Beyond regulatory T cells, histotripsy has been found to increase the number of CD8+ and CD4+ helper cells in the spleen (22, 112, 142). These changes in T cell populations in secondary lymphoid organs after histotripsy treatment implies that similar changes occur within the tumor, and that these cells are primmed against tumor specific antigens.

While all of these studies established correlative changes in T cells after histotripsy treatment, it is important to determine the efficacy and tumor specificity of programmed cells. CD8+ cells isolated from the spleens of mice that had subcutaneous colon adenocarcinoma tumors 10 days post-treatment with mHIFU were co-cultured with the corresponding cell line (140). The CD8+ T cells were found to be more cytotoxicity against the cancer cells compared to the lymphocytes isolated from mice treated with tHIFU. In this same study, ELIspot assays showed that mHIFU generated a higher magnitude of tumor-specific CD8+ cells than tHIFU (140). In a similar study with histotripsy treated prostate tumors, CD8+ T cells harvested from the spleen were found to be tumor-specific and were subsequently activated when challenged *in vitro* with the tumor cells (141). Finally, in a third study, the treatment of a known antigen into a cell line before tumor engraftment allowed for the determination of CD8+ T cells in the tumor-draining lymph nodes that were primed against tumor-specific antigens after treatment with CCH (22). Together, these studies support the hypothesis that histotripsy treatment of the local tumor is effective at generating tumor specific CD8+ cytotoxic T cells that are found systemically in distal lymph nodes and the spleen.

The efficacy of adaptive immune system activation is shown through systemic tumor killing both through the reduction of metastasis and control of contralateral tumors. Several studies have evaluated this phenomenon. For example, in a study using immunocompetent New Zealand White rabbits with VX-2 (leporine papilloma) tumors grown within a single kidney, histotripsy treatment on day 13 did not show a change in metastasis by day 19 (143). Ultimately, the authors associated the lack of difference to the aggression of the cancer (143). Beyond this study, it should be noted that the majority of histotripsy studies have actually shown significant improvements in metastasis (22, 142). For example, in a murine melanoma model, tumors were grown subcutaneously, treated with histotripsy, and amputated two days after treatment (142). For most studies conducted to date, decreases in lung metastasis are correlated to systemic cancer control and suggestive of an abscopal response. However, as an alternative interpretation of the data, the decrease in metastasis could simply be an effect of the primary tumor ablation not being able to produce as many circulating tumor cells due to its reduced size. This is a common critique of focal tumor ablation studies that report changes in metastatic burden.

Based on the current data in the field, we believe that it is likely that both interpretations are accurate; whereby, the decrease in metastasis is due to both the activation of the systemic anti-tumor immune response and the debulking of the primary tumor. Thus, it is essential that future mechanistic work in the field design experiments designed to better establish causation. For example, in addition to the prevention of additional tumor growth through reduced metastasis, there are multiple reports of CD8+ cytotoxic T cells in contralateral tumors (22, 115). To evaluate histotripsy in these contralateral models, mice were inoculated with bilateral subcutaneous B16GP33 melanoma tumors and after 10 days of tumor growth one of these tumors were treated. In the treated tumor, there was a sharp infiltration of CD8+ T cells that localized in the ablation margin, while over the course of a week the contralateral-untreated tumor slowly saw a perfuse increase in CD8+ T cells (22). This study establishes that in injected, identical tumors that a systemic effect can be achieved. As a *de novo* model of contralateral tumors, Eker rats have been deployed. Eker rats are a model with an insertion in the rat homologue of the human tuberous sclerosis gene (*TSC2*) that spontaneously develop multiple renal cell carcinoma tumors throughout both of their kidneys (144). In a study of boiling histotripsy, Eker rats had one approximately 0.5cm^3^ tumor treated and after 48hrs, their treated and contralateral kidneys were collected for immunohistochemistry staining for CD8+ cells. This found that if one tumor was treated then both tumor riddled kidneys would see an influx of CD8+ T cells, while sham treated rats did not see a change in either kidney (115). These contralateral tumors have been reported to have decreased growth rates after histotripsy treatment of the targeted primary tumor, and more significantly than radiation ablation or RFA (22). Using mice with B16GP33 tumors, after 10 days of tumor growth mice were either treated with histotripsy, RFA, radiation therapy, or sham-control treated. This study found that the mice that were treated with histotripsy saw a significant increase in tumor infiltrating CD8+ T cells in treated tumors compared to RFA and radiation ablation, neither of which saw any increase from sham-controls (22). This not only further strengthens the idea that histotripsy can stimulate a systemic immune response, but also supports the idea that non-thermal and non-ionizing therapies are more effective.

While there is a reduction in metastasis in these studies, the more established contralateral tumors only have their growth slowed, not inhibited nor reversed. This implies that while there is a systemic immune response against the cancer, it is not strong enough to prevent growth or be curative without combination with other therapeutic methods or additional treatments. Further, the efficacy of mHIFU treatment of generating systemic tumor-specific protection has been shown with the reduction in tumor growth rate in challenge tumors injected 6 days after treatment (140). Notable, the effects of mHIFU to minimize the growth rates of treated tumors were more effective than tHIFU, even though the debulking on the primary tumor was much greater (83% and 43% reduction in tumor volume respectively) (140). This indicates that the non-thermal ablation modality stimulated a stronger immune response. However, as with the pre-existing contralateral tumors, the post-treatment injection challenge still allowed for tumor growth. Together these studies reveal that histotripsy has the power to prime the murine immune system sufficiently to reduce systemic tumor burden through control of naturally occurring metastasis, spontaneous similar-primary tumors, and secondary tumors injected as a challenge.

### Combination Therapy With Checkpoint-Inhibitors

Given that many cancers are immunologically resistant to checkpoint-inhibitors due, in part, to the immunological state of the tumor microenvironment, it is critical to explore the potential of histotripsy as a strategy to improve tumor responsiveness. The increased release of DAMPs, shift in the inflammatory state of the tumor microenvironment, enhanced recruitment of immune cells within the tumor, and evidence of a systemic anti-tumor immune response all discussed above indicate that histotripsy can have local and systemic immunomodulatory effects that may be favorable for these classes of therapeutics. Based on these changes, it is reasonable to hypothesize that the shift of the tumor microenvironment to a “hot” environment should increase the effects of checkpoint inhibitors allowing for a potentially stronger anti-tumor immune response. The efficacy of anti-CTLA-4, anti-PD-1, and anti-PD-L1 therapies is correlative to the levels of expression of CTLA-4, CD80/86, PD-1, and PD-L1 in the patients’ tumor cells, APCs, and T cells (**Figure 3**). Therefore, before studying the effects of specific checkpoint inhibitors, it is critical to determine if there is supporting data to suggest that histotripsy may have a synergistic effect (76). For example, anti-PD-L1 targeted therapies are not typically used in cases where tumors do not express high levels of PD-L1. However, this is not always a clear decision, especially for checkpoint inhibitors that can be induced or up-regulated following treatment. Take for instance the expression of CD80/86 on dendritic cells, which was found to increase *in vitro* when stimulated by mHIFU supernatant, indicating that the lysate released from cells after histotripsy treatment can stimulate an upregulation of a checkpoint inhibitor (130). *In vivo*, CD86 was significantly increased on dendritic cells found in the spleen, and CD80 was increased significantly on dendritic cells in both spleen and tumor draining lymph nodes (141). It has also been shown that there is increase in PD-L1 in tumors 72 hours after treatment with mHIFU (112). As mentioned above, this could be due to the downstream effects of IFNγ production following treatment. While this is typically detrimental to the systemic immune response, these *in vivo* upregulations of checkpoint molecules after histotripsy treatment indicates that there may be an increased window of opportunity to utilize checkpoint inhibitors for synergistic therapy to potentially improve anti-tumor immunity.

With the multiple studies suggesting that histotripsy ablation can increase the expression of both CTLA-4 and PD-1 pathway receptors, it is reasonable to hypothesize that histotripsy in combination with checkpoint inhibitor therapies should have a synergistic effect. This has been explored in a pair of recent studies. In one study, individual CTLA-4 antibody therapy and CCH ablation decreased the rate of contralateral tumor growth, but there was an even more significant decrease with combined therapy in both melanoma and hepatocellular carcinoma (22). For this study, one group of mice bearing subcutaneous B16GP33 melanoma tumors were administered two doses of CTLA-4 monoclonal antibody prior to histotripsy and an additional treatment afterwards. Another group of mice bearing subcutaneous Hepa1-6 hepatocellular carcinoma tumors were given three doses of CTLA-4 monoclonal antibody prior and one post histotripsy treatment. For both of these tumor studies, melanoma and hepatocellular carcinoma, the combination of CTLA-4 and histotripsy yielded significantly reduced tumor volume compared to either treatment individually (22). In a second study, three doses of both CTLA-4 and PD-L1 antibodies in the days following mHIFU treatment of subcutaneous neuroblastoma tumors significantly increased the survival of animals compared to other therapeutic combinations (112). This study went further and performed a similar experiment, but instead of only looking at the effects of a single mHIFU treated tumor there was a second tumor grown contralaterally. In this report, after unilateral treatment with mHIFU and systemic CTLA-4 and PD-L1 therapy there was complete remission of both tumors, an effect that was not seen with any other therapy combination (112). Together, these studies demonstrate the improved therapeutic effects of the combined therapy over either histotripsy or checkpoint inhibitors independently in the murine models.

## Conclusion And Future Outlook

As histotripsy therapies have been developed over the past decade, the knowledge about the immune response has started to develop a more complete picture about the hypothetical mechanisms of action from DAMP and anti-tumor mediator release, to changes in local cellular immune populations, development of a systemic immune response, and therapeutic synergism with the inclusion of checkpoint inhibitor therapies (**Figure 5**). In sum, these studies suggest that within murine models there is a reproducible and perhaps even tunable immune effect generated by histotripsy modalities that is consistent across multiple tumor types. However, while these data are certainly exciting, the number of studies in this field are still quite limited and most are based on murine models with inconsistent tumor ablation quality. Looking towards the future, there needs to be an effort to compare the various histotripsy treatments and doses, as well as other mHIFU methods, in order to better understand the relationship between the extent of ablation in stimulating an immune response. Likewise, as these therapies begin to be utilized in human trials, it will be crucial to translate these findings into actionable results relevant to human patients. Additional basic studies and preclinical animal trials are also still needed to develop missing mechanistic insight and more translationally relevant studies are needed to ensure these findings occur outside of the typical model organisms. Despite these limitations, the benefits of histotripsy over other thermal ablation modalities in pre-clinical work suggest the potential of this focal tumor ablation therapy to induce a systemic anti-tumor immune response and therefore supports the hypothesis that histotripsy has the potential to positively impact the clinical outcomes for cancer patients.

## Author Contributions

All authors contributed to the article and approved the submitted version.

## Funding

This work was supported by the Virginia Maryland College of Veterinary Medicine; The Virginia Tech Institute for Critical Technology and Applied Sciences Center for Engineered Health; the Focused Ultrasound Foundation; and The National Institutes of Health.

## Disclaimer

The content is solely the responsibility of the authors and does not necessarily represent the official views of the NIH or any other funding agency.

## Conflict of Interest

EV has financial ties and research collaborations with Histosonics.

The remaining authors declare that the research was conducted in the absence of any commercial or financial relationships that could be construed as a potential conflict of interest.
